# Is Mental Capacity Law Law?

**DOI:** 10.1093/ojls/gqaf028

**Published:** 2025-09-10

**Authors:** John Coggon

**Keywords:** best interests, judicial role, legal adjudication, mental capacity law, philosophy of law

## Abstract

Abstract—How do you stop hard cases from making bad law? One way is to strip their determination of any distinctly *legal* reasoning, and deny that they make law at all. This article suggests that is the approach found in the Mental Capacity Act 2005 (MCA). With a focus on best interests determinations within mental capacity adjudication, the following argument challenges the sense (or otherwise) in conceiving of such adjudication as a *legal* exercise. I argue that MCA cases do not involve the courts in either a law-applying or even a law-making role. Rather, they represent the issuing of a decision that is eminently non-legal in nature, and more reflective of the exercise of an executive or administrative function. This raises questions about the quality and defensibility of mental capacity jurisprudence itself, but also about the meaning of law and the role of the judicial branch.

[Best interests] decisions when made administratively can be challenged in the Court of Protection but … such decisions made judicially are very difficult to challenge. Yet these decisions can have lifelong implications … It raises the basic questions about discretion canvassed in this book. Are we as a society aware of the powers which judges exercise in our name? Do these powers exist with our consent? Do judges in exercising them have (and deserve) that consent? The complexity of our society may mean that there are few serious alternatives to the existence and exercise of discretionary powers, but in a healthy democracy these questions should be raised and considered.^1^

## 1. Introduction

This article responds to the call in the above quotation from Sir Mark Hedley, a retired Justice of the High Court. In so doing, it radically challenges the very idea of mental capacity law and its adjudication in England and Wales. That law is enshrined in the Mental Capacity Act 2005 (MCA). The MCA aims to protect and privilege, but also limits, adults’ personal decision-making freedom in relation to their health, welfare, property and affairs.^2^ It determines how the legal assumption that a person has capacity may be rebutted.^3^ And when that assumption is displaced, it determines how and by whom decisions must then be made.^4^ I examine the MCA here by asking whether mental capacity law is law at all. The practical focus is judges’ application of the MCA’s best interests standard, which is the basis of decision making when a person is deemed to lack capacity to make their own decision. It is not the only mechanism through which a decision may be made for such a person. Nevertheless, it is the default, and it circumscribes *all* decision making where a person is deemed to lack capacity, except (ostensibly) advance refusals of medical treatment.^5^ While I do not focus on the MCA’s tests for making a determination of (in)capacity, I would argue that the following also relates to capacity assessments, building on reasons that Binesh Hass has explained well.^6^ My goal is to advance two levels of critical and practical insight. First, this article offers a framing for understanding and evaluating judges’ reasoning in and across mental capacity law adjudication. Second, and more significantly, it permits a novel perspective on and evaluation of the judicial role and the meaning and operation of the idea of law itself. The argument presents judges holding an executive or administrative role, rather than a law-applying, or even law-making, function.

It bears acknowledging that the MCA’s application mainly happens outside of the courtroom. But that fact does not lessen the importance of scrutiny specifically of the judicial role under the Act, and this article’s focus is on the function of judges under the MCA. It also bears acknowledging that a sharp response to the question ‘Is mental capacity law law?’ might just be ‘Necessarily it is!’. As outlined in the next section, there is an established body of common law and, in the MCA, a duly enacted statute that consolidate jurisdiction across health, welfare and property and affairs. The MCA establishes a specialist court—the Court of Protection (CoP)—to supervise that jurisdiction.^7^ There is, furthermore, a huge library of case law that has developed within the CoP and higher courts since the MCA came into force (for the greater part) in October 2007. Additionally, the MCA has been amended following legal challenges and wider scrutiny concerning deprivations of liberty; that is, it has been amended better to assure its compliance with standards enshrined in (domestically enforceable^8^) human rights law.^9^

However, to dispose of the question ‘Is mental capacity law law?’ so quickly would be too summary a dismissal. The question opens up new lines of critical scrutiny of adjudication under the Act, and more widely. It exposes significant challenges to the coherence and wisdom of formulating points of personal decision making, within the framework of the MCA, as *legal* questions. By legal questions, I mean matters that may be resolved through the expert application of legal method and institutional authority and process that distinguish judges from other decision makers. The less ‘law-like’ we find mental capacity jurisprudence to be, I argue, the more anxiously we need to explore the question raised in this article. And this extends its relevance within legal theory more widely. Generally, philosophical analysis of legal adjudication focuses on questions concerning courts’ institutional functions as regards law-applying and law-making powers. However, as already indicated, I am inviting us to look to the courts’ assuming an *executive* rather than a legal-interpretative or legislative function. Within the article, I limit my analysis to MCA jurisprudence, but the implications of the argument have an important and wider reach.^10^ The article first gives a brief outline of mental capacity law. I then provide my framing of law and adjudication against broader debates on these, before focusing in on MCA jurisprudence. The final two substantive sections of the article look to consequent critical matters—in relation to the powers of judges under the MCA, but also to understandings of law itself.

## 2. Mental Capacity Law in England and Wales

The jurisdiction created through the MCA has significance of both enormous breadth and depth. As Sir Mark indicates in the above quotation, judges’ powers under the Act are tremendous, and represent the final determination that may be made on a given matter of personal decision making. Judges can make the concluding call, for instance, on life-and-death decisions, on questions such as where a person will live or with whom they may associate, on how a person’s money will be spent, on the imposition of controversial and unwanted medical interventions, on sexual and reproductive freedoms, and so on.^11^ Contemporary mental capacity law rests on the continuation of an ancient jurisdiction.^12^ Its current form and institutional arrangements may be seen as a culmination of judicial activism, statutory consolidation and consequent (and ongoing) developments in practice across the past four decades. This period has seen mental capacity law track interweaving dynamics in sociopolitical (including legal) norms, economic situations and cultural developments. These cover, for example: changing understandings of disability and disability rights; changes in public law adjudication and the arrival of the Human Rights Act 1998; changing roles and expectations of government (including local authorities); developments in healthcare administration, practice and rights; and evolving norms and expectations in the legal supervision and scrutiny of different professions (such as the retreat from the courts’ earlier, highly deferential approach to the views of medical professionals). It is impossible to capture the subtlety and effect of such developments succinctly, less still whilst conveying clear contextual understanding and their relationship with legal scholarship on mental capacity law as this too has developed. Peter Bartlett has recently published a paper that addresses that task at good length.^13^ For the purposes of the current article, I much more briefly present key points about the law’s recent development, allowing a view of what it is designed to do, and how.

The MCA was enacted following a lengthy process consequent to a Law Commission review.^14^ That review surveyed a situation in which the Crown’s *parens patriae* jurisdiction had been removed through the Mental Health Act 1959, and replaced by statutory governance that had in turn been reformed under the Mental Health Act 1983. This provided a framework for decision making regarding property and affairs, supervised under the jurisdiction of the previous Court of Protection. As regards questions concerning health and welfare, however, there was limited statutory provision. Most emphatically, there was an absence of legal power for third parties to make decisions where an individual was, by reason of a determination of incapacity (itself not clearly defined at law), legally unable to make a decision on their own behalf. These limitations were identified as a gap in the law. In 1989, in *Re F (Mental Patient: Sterilisation)*,^15^ the Appellate Committee of the House of Lords introduced the best interests standard as the legal mechanism for adjudging the lawfulness of an intervention for a person deemed to lack capacity—an intervention that would otherwise find no lawful basis. In that case, it rendered permissible a non-emergency, non-therapeutic medical intervention—the sterilisation—of a woman who lived in an institutional care setting. In Lord Brandon’s words, the basis of the decision reflected the recognition ‘of first importance that the common law should be readily intelligible to and applicable by all those who undertake the care of persons lacking the capacity to consent to treatment’.^16^ In unpacking the point, Lord Brandon emphasised that it would be ‘intolerable’ for health and care professionals to face a ‘dilemma’ of being duty bound to serve their patients’ best interests, whilst also suffering the threat of being found liable for trespass to the person.^17^

Against this background of absent or unclear law, and the perceived role for a legal best interests standard as the solution to it (by providing legal security for the benefit of decision makers), in the decade and half that followed *Re F*, two parallel activities occurred. One was the Law Commission review and then parliamentary creation of what became the MCA. The other was a phased legal evolution in the courts to address the gaps identified in the law. This resulted in a common law test for (in)capacity and a common law framing of the best interests standard.^18^ These were very much parallel activities, with the courts’ framing and understanding mirroring closely what came to be proposed by the Law Commission and subsequently enacted by Parliament. The common law (in)capacity test was ‘functional’, checking the person’s ability to make a given, specific decision: could the person understand and retain (what was deemed to be) the relevant information, believe it and weigh it up in arriving at a choice?^19^ As regards best interests, judges provided a sparsely defined idea. Initially, its substantive content may be characterised as doubly empty: as well as there being scant positive definition of what the standard meant, it was one on which judges showed great willingness to look beyond their own competence for an applied, expert determination of a particular best interests decision.^20^ And beneath this there was conflicting judicial statement both on the right approaches to making a best interests decision and on the fundamental legal basis of the standard itself.^21^ Over time, best interests evolved—notably, more firmly to articulate and centre distinct insights that judges could bring.^22^ But it remained lightly specified within the domain of health and welfare jurisdiction.

Alongside evolving common law, the MCA was developed and drafted as a principles-based piece of legislation. As noted above, it drew together jurisdiction of health and welfare with that of property and affairs, and created a new CoP to supervise the jurisdiction that it created. The MCA’s principles are designed to promote and protect the starting point that at law an adult is assumed to have capacity.^23^ It provides procedural steps that must be taken before that assumption may be displaced, with a functional test for (in)capacity that slightly differs from that at common law.^24^ And for the event that a determination of incapacity is made, the decision-making standard remains best interests.^25^ That standard, which is the practical focus of the current article, is detailed in MCA, section 4: a provision that offers a non-exhaustive, non-ordinally ranked ‘checklist’ of factors that a decision maker (D) should take into account when determining what would serve the best interests of a person who lacks capacity (P). These factors include P’s past and present wishes and feelings, and the beliefs and values that P would bring to bear on the decision if they had capacity.^26^ But the provision is expressly not just about seeking to find and honour the decision that P would make, even should that be perfectly discernible. The Act requires D to ‘consider all the relevant circumstances’,^27^ which include both circumstances ‘of which the person making the determinations is aware’ and those ‘which it would be reasonable to regard as relevant’.^28^ The Act gives no complete or even strongly directive account of what to lend weight to, or how much weight to lend it, in arriving at a decision. As explained below, it is, by design, a skeletal, non-comprehensive source of guidance for decision makers. Such further direction as there is beyond the Act itself—notably the Explanatory Notes to the Act and the statutorily created Code of Practice—provides indications and examples rather than narrowing principles or detail.

It is that body of law that this article scrutinises.^29^ In closing the overview, it is important to stress that since the MCA came into force, an enormous body of case law has materialised.^30^ This (unsurprisingly) primarily emanates from the CoP, but there are also significant appeal court decisions. As regards the number of cases, just in relation to health and welfare, ahead of the enactment of the (then) Mental Capacity Bill, the government anticipated 200 cases a year.^31^ As Lucy Series and colleagues discovered, the actual volume has proven to be vastly greater: in 2017, they showed that in 2016 there were over 4000 welfare-related applications to the CoP, with that number expected to increase further.^32^ From the MCA caseload, as Bartlett explains, trends and overarching practical developments are observable.^33^ Regarding trends, there is, for instance, a greater emergence of gravity in relation to P’s subjective wishes, feelings, beliefs and values; a gravity that emanates notably from the Supreme Court decision in *Aintree University Hospital NHS Foundation Trust v James*, but whose less than compelling nature (which I revisit below) underscores the point here.^34^ Regarding overarching practical developments, there is an expansion in detail and form regarding questions of legal procedure. However, in what follows, I suggest that, notwithstanding an abundance of judicial activity, there is good reason to question how much substantive *law* actually issues from the MCA or judicial determinations made under it. To explain this, I next provide a general framing of law and adjudication, and judges’ exercise of discretion, before focusing on why we might perceive mental capacity law as a notable, and potentially problematic, outlier.

## 3. Law, Adjudication and Judicial Discretion

Within this section, before directing my attention specifically towards mental capacity law, I characterise general ideas of law and legal adjudication. Any account of these will invite contest. But to provide as apt and widely acceptable as possible an account of law and the function of judges, I stipulate conditions that are very limited in their conceptual and practical content and demandingness. They speak to necessary criteria for recognising norms as *legal* norms, whilst leaving open the substance, basis and scope of further conditions that might *in addition* be applied according to distinct and diverse philosophical claims about the meaning of law. They also, when I come to discussing adjudication, leave the detail open on the basis and boundaries of *extra-legal* reasons that may feature in judgments. Specifically, I draw from the conceptual framing of Joseph Raz, whose contributions to the understanding of legal positivism provide outstanding conceptual rigour to analyses of law and legal doctrine, including for theorists whose analyses incorporate understanding of positive law even while allowing for a broader reach for the overarching concept of law than a purely legal positivist approach would permit.^35^

As such, I hope that my approach permits a framing of ‘law’ that may be recognised as incorporated across (of course not all) differing, mutually contradictory, wholesale theories of law and of adjudication. Also of importance, the conditions are limited in how taxing they are in applied settings. That is, they allow for real-world considerations and limitations in their application, rather than some sort of ideal-type imagining of how they should work in some perfect, abstract, imagined court. And in doing that, they speak to systems of social, political and legal norms and institutions within common law jurisdictions such as England and Wales. Finally, as well as providing an explanatory framework, they create a useful measure for critical evaluation.

### A. The Capability for Effective Action Guidance as a Necessary Component of the Idea of Law: Towards an Understanding of Law, Adjudication and Judicial Discretion

#### (i) Formalist accounts of the rule of law as a conceptual starting point

I begin addressing the meaning of ‘law’ for the purposes of this article by drawing from ‘thin’, formalist accounts of the rule of law.^36^ Such accounts provide criteria for a normative system to do what a specifically *legal* system must aim to do. In taking this approach, I do not conflate the rule of law with a wholesale concept of law. Rather, the value here comes as the sorts of criteria characterised in thin accounts of the rule of law are, within a practical legal system, necessary conditions of something being described as ‘good’ law. They are minimal essential aspects, amongst potential other, much wider ones, that the designation ‘law’ might also imply. For its clarity and cogency, I draw from Raz’s essay ‘The Rule of Law and Its Virtue’.^37^ There, Raz provides two groups of indicative principles that a formalist idea of the rule of law entails. He does not intend that his enumerated principles be considered a comprehensive list. Nevertheless, he is clear in saying that ‘in the final analysis *the doctrine rests on its basic idea that the law should be capable of providing effective guidance*’.^38^ That ‘basic idea’ sits at the heart of, and circumscribes, whatever more specific principles are identified as aspects of the rule of law. As Raz argues, effective action guidance is the focus because legal governance (my term here) relies on the idea that people should obey law, and that they are able to do so. In Raz’s words: ‘if the law is to be obeyed *it must be capable of guiding the behaviour of its subjects*. It must be such that they can find out what it is and act on it.’^39^

As just indicated, Raz presents two groups of ‘the more important’^40^ principles that the rule of law generates. The first group, encapsulated in his first three principles, concerns effective action guidance. The second group, encapsulated in the remaining five principles, concerns the need

to ensure that the legal machinery of enforcing the law should not deprive it of its ability to guide through distorted enforcement and that it shall be capable of supervising conformity to the rule of law and provide effective remedies in case of deviation from it.^41^

Raz’s list of eight principles (on each of which he expands within his essay^42^) is as follows:

(1) All laws should be prospective, open, and clear.

(2) Laws should be relatively stable.

(3) The making of particular laws (particular legal orders) should be guided by open, stable, clear, and general rules.

(4) The independence of the judiciary must be guaranteed.

(5) The principles of natural justice must be observed.

(6) The courts should have review powers over the implementation of the other principles.

(7) The courts should be accessible.

(8) The discretion of the crime-preventing agencies should not be allowed to pervert the law.^43^

As contrasted with the wider organs of government, in relation to the judicial branch and the rule of law, Raz notes specific and distinct limitations against the exercise of arbitrary power: ‘The one area where the rule of law excludes all forms of arbitrary power is in the *law-applying* function of the judiciary where the courts are required to be subject only to the law and to conform to fairly strict procedures.’^44^This is important to the analysis that follows. But it is straightforwardly the case that the judiciary’s law-applying role does not exhaust its functions. Raz’s claim here does not speak to judges’ *law-making* functions—a point that he notes expressly.^45^ Some discussion is thus needed of adjudication, allowing that we cannot limit inquiry through an analysis that restricts the judge’s role to the narrow application of legal method.

#### (ii) Common law adjudication: applying law and making law

In arguing in this article that mental capacity law adjudication is radically distinct, I do not deny the inevitable place for discretion, and the inevitable application of contestable (non-legal) values, within the judicial role. For one thing, judges’ discretion is a signal feature of a common law system: judges within such a system possess a law-making as well as a law-applying role. Of course, against that bare observation are a range of analyses to describe, explain and justify or challenge what that discretion does and should mean—or whether ‘discretion’ is the wrong word to use.^46^ In relation to the exercise of (what is often framed as) discretion, it is widely recognised that it applies where there is vagueness or open texture in the language of statutory or common law rules that create uncertainty about specific application;^47^ for instance, in tests for reasonableness or foreseeability. It is also widely recognised that novel situations arise that create problems of legal indeterminacy, whether the novelty concerns something such as a radically distinct new technology or technique;^48^ a change in judicial recognition of social acceptance that renders the continued application of anachronistic but existing legal rules intolerable;^49^ or a change in the gravity of the direction in which individual legal norms collectively pull.^50^ Furthermore, we find broader challenges within legal method, for instance as regards the possibility (or otherwise) of identifying a case’s *ratio decidendi*, or the clarity in drawing a line between distinguishing a case and departing from an existing legal rule.^51^ Whilst profound differences are found in framing and scrutinising these points, they are a recognised feature of common law judging within legal philosophy,^52^ socio-legal studies^53^ and, indeed, debates and commentaries by and with judges themselves.^54^

In short, the application of discretion is an essential and inevitable aspect of the judicial role; a discretion that requires the invocation and application of values that are not straightforwardly or uncontroversially determined through a review of existing points of positive law. In this broad encapsulation, judges hold their own, distinct *legislative* function. At times, they create a legal norm, rule or principle. And such practice invites various sorts of critical scrutiny. In legal theory, that ranges, for example, from concerns about retroactive application of legal rules to the possibility of legal certainty within a system that promulgates general rules whose particular application may have (whatever levels) of inherent uncertainty. It invites ‘boundary’ disputes, perhaps most strikingly in constitutional and public law literatures that ask which questions are apt for legal—as opposed, for example, to political—resolution. And in practice, critical scrutiny looks beyond philosophical identification of discretion’s place and its role to points of (conscious or unconscious) bias having a determinative effect within judging and the demonstrable place of particular judges’ values within their approach to arriving at decisions.

Nevertheless, and crucially for the current article, the focus this places on judges retains these questions within a system of *legal* decision making, even if it involves scrutiny of judges’ own (potentially unjustified) *making* of the *law* before their application of it. Accordingly, even a position such as one embodied in legal realist theory allows for the trappings of legal form to be honoured, however disingenuously, within the judicial function.^55^ But importantly, less sceptical schools than legal realism allow that within adjudication there is some sort of rigorous and robust core of ‘the legal’ at the heart of the judicial role. Insofar as this is accepted (as it were) on the judiciary’s own terms, it is accounted for by reference to a judge’s possession of practical wisdom; a point to which I now turn.

#### (iii) Common law adjudication: applying law whilst exercising wisdom

Common law judges are not simply legal experts, nor is there a pretence on the part of the judiciary that legal expertise is all that judges should bring to their role. In mediating the boundaries of how and when to apply a point of law, or in establishing when to amend or otherwise create new law, judges are expected to be possessed of and to exercise practical wisdom. This is reflected in Raz’s description of expectations of judges’ skills and character:

When discussing appointments to the Bench, we distinguish different kinds of desirable characteristics judges should possess. We value their knowledge of the law and their skills in interpreting laws and in arguing in ways showing their legal experience and expertise. We also value their wisdom and understanding of human nature, their moral sensibility, their enlightened approach, etc. There are many other characterisations which are valuable in judges. For present purposes these two kinds are the important ones.^56^

Such a point bears spelling out because of how it emphatically contrasts the application of moral judgment with what might be labelled the blunt application of legal skill or argument. It is a qualitatively distinct function. In its presentation, Raz’s framing here carries a similar sentiment, but distinct categorisation, to representations that insist on all such skill being embraced by an accordance with law. For instance, former Lord Chief Justice, Lord Judge, frames the same idea as follows:

We make great demands of our judges. They must have wisdom, patience, a sense of practical realities, an understanding of people and the way of the world, fairness and balance, independence of mind and knowledge of the law, and a total commitment that justice should be administered according to the law.^57^

The subsumption of this application of practical wisdom *within* ‘law’ is also found in the characterisation of the judicial role on the UK judiciary’s website:

In a democracy, it is of vital importance that the public and those who appear before judges trust that their cases will be decided in accordance with the law. This can only be achieved if judges and the judiciary as a whole are independent of external pressures and of each other. For judges to discharge their constitutional responsibility of providing fair and impartial justice, it is solely relevant facts and law that should form the basis of their decisions.^58^

Within discussions of legal theory and practice, the conceptual site of contest here finds a famous provocation in Lord Reid’s paper ‘The Judge as Law Maker’.^59^ Jurisprudential debates have seen efforts to reconcile apparent incongruities by distinguishing (apparent) rules of positive law and a wider, overarching normative system that is ‘the law’, with judges’ application of wisdom manifesting in constraining and amending the former by application of the latter.^60^

I do not seek to settle the conceptual dispute that arises here. Within common law adjudication, suffice to say that if we want to address questions concerning the judicial function with reference just to operation within ‘law’ as a normative system, we must capture questions of judicial discretion and judges’ law-making function under a label of ‘law’ that embraces (at least) two qualitatively distinct concepts. First, we have (sufficiently) identifiable, positive laws. Second, and distinctly, we have an overarching, morally attuned normative system that reflects moral ideas of justice as part of (something like) the ‘essence’ or ‘spirit’ of law. Alternatively, we may more bluntly say that there is positive law, and then there is regard to extra-legal ideas of justice that also account for what is entailed in the judicial role. Either way, within the process of adjudication there are necessarily at least partial legal inputs in the form of identifiable law(s) to be applied, and an output that is identifiable as the affirmation or generation of law—a source of practical normativity that may effectively and bindingly guide other action in future.

In other words, in exercising discretion, at a minimum, a judge’s exercise of wisdom governs the scope to identify and then to apply or disregard a positive point of law, ie *some* practical legal reasoning needs to be describable. And where there is the generation of new law—whether through more modest or particular refinement, or something more generally reformative or radical—it is emphatically *positive law* that is issued, to guide and indeed bind future action. My argument in this article is that something very distinct happens in mental capacity law: that no positive legal content determines the decision; *and* what is issued as a decision is not law. Rather, a judge’s wisdom (such as it may be) itself becomes the standard that is reflected back through a judgment; and with it, the substance of a judge’s best interests determination becomes a wholesale exercise (or an attempted one) in applied wisdom rather than applied law.

### B. Adjudication, Judicial Discretion and a Focus on the Court of Protection: Three Warning Bells as the Place of Law Diminishes

The above analysis offers a general characterisation of law and adjudication. It permits that judges operate within boundaries that extend beyond simple identification and application of ‘pure’ legal reasoning. I have *not* suggested that ordinarily judges operate only with regard to distinctly legal considerations. The judicial role calls for the exercise of discretion involving values that are extra-legal, or that are not anyway established or clear determinations drawn from existing points of positive law. In the remainder of this article, I will explain and critically engage with the view that, even accepting that point, mental capacity law adjudication should not be taken as a version (even an ‘extreme’ version) of ‘judging as usual’ or ‘judging within the bounds of the usual’. My argument is that we have a problem with and for mental capacity law because it is categorically wrong to suggest that the courts are either applying law or acting in a law-making role. As a bridge to that, I close the current section with three sources of reflective caution to support the argument.

#### (i) A warning bell from legal theory

In explaining the special nature of the courts as institutions that use law and legal reasoning as the distinct basis and method for resolving disputes, Raz presents a hypothetical counter-example (intendedly hypothetical, however prescient it might come to seem!). This is used to demonstrate and explain what is distinctive about the judicial function and a court of law’s claim to authority. A reader can take the following without needing to accept Raz’s overarching jurisprudential positions. It is not a short quotation, but I provide it in full because it provocatively sets up an apt framing for analysis of adjudication in mental capacity cases:

Consider a normative system containing only rules instituting tribunals and regulating their operation. When a dispute is referred to a tribunal it will authoritatively be settled by its decision, for the system includes a rule to that effect. It contains a rule making it obligatory to observe the decisions of the courts and providing for their enforcement by a police force created for this purpose only. In such a system the courts can settle any dispute in any way they like. There are no legislated, customary, or any other standards which they have to apply. Nor do they have to follow their own precedents. The courts of this peculiar system are not entitled to decide in an arbitrary way. They are instructed by a rule of the system to make that decision which seems to them the best in the circumstances, taking account of all the considerations which seem to them relevant. Since the courts have to act on reasons and reasons are general, we could expect some regularity in the decisions of the courts. The same judge hearing two very similar cases on the same day is likely to reach the same decision in both. But cases will be heard by many different judges and judges may change their mind as well as forget complex and intricate arguments, etc. Consequently, the decisions of the courts over a period of time are unlikely to reflect any consistent line on any one issue. The degree of regularity will depend on contingent factors such as the number of judges, the degree of uniformity of their social background, etc.^61^

Raz opines that ‘It is unlikely that such a system has ever existed or will ever exist’.^62^ His purpose in providing the example is to tease out the distinction of such a system as contrasted with one that takes and applies *law* and *legal* reasoning. As I will argue below, the jurisdiction that the MCA creates—including with it the CoP—is little removed from Raz’s characterisation of something that is at once court-like but eminently not a court of law.

#### (ii) Warning bells from early medical best interests cases

In the second section of this article, I gave an overview of modern mental capacity law as it emerged and developed. As noted there, for health and welfare cases, the decision in *Re F* was pivotal. In that case, there was no question or pretence about the application of existing law: the court was responding to an absence of legal rules permitting non-emergency medical interventions for adults who were (deemed) unable to give or refuse consent (or put otherwise, responding to the overbearing presence of legal rules rendering such interventions plain unlawful). In establishing the best interests standard as the basis of lawful decision making for otherwise non-consensual battery of such adults, the House of Lords gave astonishingly little detail on what the standard actually meant. In a contemporary review of the case (with the subtitle ‘Judges Rule OK?’), Josephine Shaw argued that:

It is to be regretted … that in the interests of legal certainty, no identifiable criteria have been adopted to regulate the exercise of both medical and judicial discretion. In the long run, no other solution will eradicate the possibility of abuse.^63^

Consistent with broader judicial approaches at the time, *Re F* may be characterised in part by reference to its medical context: it is well viewed within a history of judicial deference to the medical profession (as notably reflected in the law of negligence at the time^64^). But for the purposes of the current article, questions of ‘medicalisation’ or deference to doctors are not centrally important.

The important point to take from Shaw’s critique is how it sits in relation to the ideas presented above in relation to the rule of law. She identifies problematic legal uncertainty, and how this combines with a critical focus on the (in)aptness of the application of judges’ power.^65^ For Shaw, that is in part because of the limits inherent to the courts as a particular sort of institutional decision maker. As indicated by her words above, it is in part too because what the courts did provide was a remarkably empty and unclear standard. But it is also a question of judges’ competence given the skills and expertise required to make a best-interests-type decision. Tellingly, that last point was not just a concern from critical legal commentators at the time. Shaw headed her paper on *Re F* with a dictum from a Canadian case, citing Justice La Forest in *Re Eve*:

Judges are generally ill-informed about many of the factors relevant to a wise decision in this difficult area. They generally know little of mental illness, of techniques of contraception or their efficacy. And however well presented a case may be, it can only partially inform.^66^

Such reasoning can also be found within the English law reports. For instance, soon after *Re F*, in *Airedale NHS Trust v Bland*—a life-or-death case where the House of Lords tried (perhaps a bit too hard) to articulate rationalisations of the best interests standard—Lord Mustill held that:

[I]f the law requires the decision-maker to consider whether a certain course is ‘in the best interests’ of the patient, the skill and experience of the judge will carry him only so far. They will help him to clear the ground by marshalling the considerations which are said to be relevant, eliminating errors of logic, and so on. But when the intellectual part of the task is complete and the decision-maker has to choose the factors which he will take into account, attach relevant weights to them and then strike a balance the judge is no better equipped, though no worse, than anyone else. In the end it is a matter of personal choice, dictated by his or her background, upbringing, education, convictions and temperament.^67^

In short, insofar as best interests was established as (and has, of course, remained) a wide-open legal standard on its own terms, both critical legal commentators and judges themselves sounded warnings against the sense of pitching it as well determined through the application of *legal* skill. Where it is to be addressed by a judge, it has been accordingly recognised as a standard to which judges will bring their own individual views, rather than the application of legal method.

#### (iii) A warning bell from a retired judge’s call for scrutiny

I have headed the current article with a quotation from Sir Mark Hedley’s book *The Modern Judge*. For its candour, balance and humble and productive invitation to scrutiny, I consider that book to be an essential read for anyone interested in mental capacity law or in the judicial role. When considering best interests decision making under the MCA, Sir Mark does not say that the Act provides for an unfettered discretion. He is, however, centrally concerned with the enormous breadth of discretion afforded to judges, and the (lack of) limitations on that discretion. As explained above, section 4 MCA does not present an exhaustive checklist of matters to consider, offers no hierarchy or presumptive ranking across the different considerations that it does list and is not indicative of the answers that it aims to provide. This means, says Sir Mark of judicial determinations, that ‘In practice the discretion, if not actually unfettered, is in truth very wide’.^68^ Again as noted above, section 4 MCA requires that ‘all the relevant circumstances’ be considered,^69^ with section 4(11) explaining that:

‘Relevant circumstances’ are those—

(a)of which the person making the determination is aware, and

(b)which it would be reasonable to regard as relevant.

As Sir Mark comments, even if the judge’s decision about best interests is not unfettered, ‘You cannot get wider than that’.^70^

Such points tie in, for Sir Mark, to the following reflection:

The discretionary powers of the modern judge are seen most vividly—and most controversially—in the areas of child welfare and the best interests of those unable to make their own decisions. The question is: are discretionary powers compatible with justice? In his seminal work ‘The Rule of Law’, the late Lord Bingham wrote: ‘Questions of legal right and liability should ordinarily be resolved by the application of the law not the exercise of discretion’. He concludes his chapter entitled ‘Law not Discretion’ with these words: ‘… No discretion should be unconstrained so as to be potentially arbitrary. No discretion may be legally unfettered.’ As it happens, he does not discuss family or mental capacity law, but their practice, superficially at least, does not always sit easily with the principle that he identifies: one with which most would want to agree.^71^

Elsewhere, in a lecture delivered in 1990 and published in a collection of his essays, Lord Bingham does address discretion within family law. The lecture’s central purpose was to rebut jurisprudential theorists’ claims that common law judges possess and exercise wide-reaching instances of discretion. Within that argument, Lord Bingham concedes: ‘Perhaps the last real stronghold of almost unreviewable discretion is where the care and custody of children are concerned.’^72^ He goes on to reflect:

It is not perhaps very happy that an unfettered right of appeal should be effectively abrogated by judicial decision, nor that, in a field where judicial decisions have a unique capacity to cause lasting misery, the trial judge’s decision should be effectively final. On the other hand, it would be very hard indeed to suggest any guideline to govern the exercise of this discretion which was not either so obvious or so heavily qualified as to be futile. It would seem that in this limited field, for better or worse, reliance must be placed on the trial judge to show the wisdom, sensitivity and insight of Solomon, although lacking the latter’s extra-judicial powers.^73^

This observation and analysis—and even rhetoric—are telling in relation to the current article’s argument. The influence of the High Court and then the MCA have considerably expanded the reach of the ‘limited field’ that Lord Bingham identifies, both in the exponential growth of adjudication on questions concerning the health and welfare of adults and in the conjoining of such jurisdiction in relation to adults’ property and affairs.^74^

In combination with the two other ‘warning bells’, as I have characterised them, these concerns amount to cause for considered critique of adjudication in this domain. What *legal* fetters apply to judges’ application of the best interests standard? And if none or vanishingly few, in the different ways alluded to across these three sources of critical reflection, how should we understand and evaluate the place, role and (socio-ethical) legitimacy of the jurisdiction created by the MCA? I explore these questions, respectively, in the final two substantive sections of this article.

## 4. Law and Mental Capacity Law

In this section, I focus on three aspects of mental capacity law that mark radical departures from meaningful understandings of law and legal adjudication. First, I consider radical judicial disagreements on fundamental questions concerning mental capacity law itself. Then, I focus more fully on the radically non-prescriptive nature of best interests decision making. Finally, I look to the radical departures in applied legal method that circumscribe adjudication under the MCA. Any of these alone may not render mental capacity law conclusively as an outlier; as problematically ‘unlaw-like’. But I argue that in combination they rob mental capacity law of too many vestiges of legal form, and leave us in a situation where judges are not applying, or even making and applying, law.

### A. Radical Fundamental Disagreement

It might be suggested that something at least approaching sufficient legal certainty may be found in MCA jurisprudence because of the enumeration of principles in section 1 MCA. It might be claimed, furthermore, that these are bolstered by (what might be termed) MCA-cultural principles that emanate from its case law and then steer decision making, within the surrounding legal constraints of common law principles such as the sanctity of life and the CoP’s obligations under the Human Rights Act 1998. Judges may protest, taking here the words of MacDonald J, that their exercise in arriving at a best interests decision is ultimately made ‘by reference to the principles … grounded in the Mental Capacity Act 2005 s.4’.^75^ And perhaps most notably as a unifying theme, appeal might be made to the ethos of so-called ‘P-centricity’ within best interests decision making following the Supreme Court decision in *Aintree v James*.^76^

However, contrary to such positions, I would argue that we do not even find fundamental agreement on how the MCA should be understood and applied, and the scope for principles from beyond the statute to determine a decision themselves belie a clear detachment from an entrenchment in firm legal underpinnings. We may note first Lady Hale’s observation in *Aintree v James* that: ‘The courts have been most reluctant to lay down general principles which might guide the decisions. Every patient, every case, is different and must be decided on its own facts.’^77^ Such variance in determinative principles may be considered particularly problematic as regards the question of whether mental capacity law is law. The MCA rests, following section 1, on principles that may stand in conflict with one another. In this sense, we may contrast it with the distinct best interests standard that governs decisions regarding children. While that also opens up questions of wide judicial discretion (and may be subject to comparable critique to that advanced in this article^78^), that jurisdiction at least on its face seeks resolution of competing arguments through the single principle of welfare. Mental capacity law, by contrast, functions against a spectrum of competing and mutually incompatible principles. This is widely presented as being between two potentially antagonistic values: autonomy versus paternalism (or protection).^79^ Yet, in practice, as well shown by Camillia Kong, even this framing is too reductive, both on its own terms and in its exclusion of a much broader network of principles, which may be derived from a complex expanse of sources.^80^

The point here may be underscored by viewing the practice within the CoP of identifying *alternative*, and still non-comprehensive, best interests checklists, including the principles within them. Theis J indicates this phenomenon’s manifestation in the case of *NHS North Central London Integrated Care Board v Royal Hospital for Neuro-Disability and another*, observing that ‘A number of cases have sought to provide a helpful non-exhaustive list of issues requiring determination in an application of this kind’.^81^ Her Ladyship specifically then identifies the approach of Cobb J in the case of *PL v Sutton Clinical Commissioning Group and another*,^82^ in which he enumerates eight factors to address in arriving at a best interests determination.^83^ These factors span PL’s previous views and the views of her family and carers. They also cover PL’s quality and enjoyment of life, and distinct prognoses, and include two substantive principles that do not feature in the MCA at all (at least in terms): ‘PL’s dignity’^84^ and ‘The sanctity of life generally’.^85^ In other words, Cobb J produces a further, distinct, still open-ended ‘checklist’ for making the decision. In doing this, he draws both from and beyond principled considerations written into the MCA.

The insecurity of fundamental principles is compounded further by the (probably inevitable, and anyway real^86^) consequences of the MCA conjoining the distinct practical and normative heritages, respectively, of the previous Court of Protection, with its jurisdiction over property and affairs, and the Family Division, with its jurisdiction over health and welfare decisions. The fundamental principled and philosophical tensions to which that gives rise are evidenced in clashing judicial characterisations of the MCA itself. From, as it were, the perspective of property and affairs, Lewison J explains in the case of *Re P* how and why the MCA ‘marks a *radical change* in the treatment of persons lacking capacity’.^87^ Baker J, by contrast, in a medical case in which he draws approvingly from Lewison J’s reasoning in *Re P*, speaks to how ‘the MCA has *refined* the previous law’ while ‘The basic principles, however, *remain unchanged*’.^88^ It might, of course, be remarked that such tension would need to arise if the MCA were to be successful in its exercise of consolidating jurisdiction. However, the burden of that success is belied by the determinations in the respective cases. The reasoning in Lewison J’s judgment pulled the best interests standard *away* from that which was gaining wider momentum in health and welfare cases, ie seeking to accommodate more and better the wishes, feelings, beliefs and values of the person about whom a best interests determination was being made.^89^

In addition, we even see radical disagreement on the in-principle potential (in some ideal-typical sense) of the best interests standard. Baroness Hale articulates a position found in the pre-MCA common law,^90^ stating in *Aintree v James* that in relation to a best interests determination ‘there can only logically be one best option’.^91^ As well as this not being straightforward philosophically,^92^ it grates against observations such as Jackson LJ’s that ‘The cases which arise for decision under Part 1 of the MCA … tend to be acutely difficult, not admitting any obviously right answer’.^93^ It might also be noted that the point that Lady Hale emphasises from pre-MCA common law comes from Dame Elizabeth Butler-Sloss P’s judgment in *Re S (Adult Patient: Sterilisation)*,^94^ where the president was correcting a claim to the contrary by Hale J (as she then was) in *Re S (Hospital Patient: Court’s Jurisdiction)*.^95^ Strikingly too, within her judgment in *Aintree v James* itself, Baroness Hale says in terms that she might have come to distinct conclusions to those reached by Jackson J at first instance, even with both being ‘correctly directed … as to the law’.^96^

Finally, the point about fundamental disagreements on the MCA presents itself in outright contradiction within different judges’ approaches to weighting and then balancing different factors to include within a best interests determination, and their distinct representations of questions of incommensurability across different values.^97^ For example, Hayden J identifies fundamental challenges in the weighting and weighing exercise of two principles, neither of which emanates (in terms, anyway) from the MCA. In explaining this, he reflects on a zero-sum clash between ‘the “inviolability of life” as an ethical concept’ and ‘an individual’s right to self determination or personal autonomy’.^98^ Pitted against one another, he characterises ‘a balance of opposites: the philosophical against the personal’.^99^ Even conceptually, this contrasts with Charles J’s presentation, in *Briggs v Briggs*, of the conflict between ‘the fundamental and intensely personal competing principles of the sanctity of life and of self-determination’.^100^ In short, we do not find disjuncture only in the identification of different principles, but even in their respective natures as principles in the CoP or their scope for reconciliation. Overall, the points raised here do not just reflect the sorts of philosophical or sociopolitical differences that we might expect to see within a judiciary comprised of distinct people. They belie the lack of a secure foundation to what is, as explained next, a radically empty legal architecture provided in the MCA.

### B. Radical Non-prescription

Perhaps the most obvious problem with the MCA as a source of clear action guidance is its radically non-prescriptive nature. In Lady Hale’s words: ‘The Act gives some limited guidance.’^101^ This idea of the MCA only giving ‘limited guidance’ has become something of a mantra, repeated in CoP and Court of Appeal judgments.^102^ In a very real sense, it provides the CoP with an uncanny resemblance to Raz’s imagined non-court, which is ‘instructed by a rule of the system to make that decision which seems to them the best in the circumstances, taking account of all the considerations which seem to them relevant’.^103^ Section 4 MCA, as we have seen, provides more than this statement; but only through a non-exhaustive guide (that, as shown in the previous section, is replaceable with varying alternative non-exhaustive guides), and with practically little extra of substance.

That it is merely an incomplete guide is reinforced by the explanatory notes to the Act, which say:

Given the wide range of acts, decisions and circumstances that the Act will cover, *the notion of ‘best interests’ is not defined in the Act*. Rather, subsection (2) makes clear that determining what is in a person’s best interests requires a consideration of all relevant circumstances … Best interests is not a test of ‘substituted judgement’ (what the person would have wanted), but rather it requires a determination to be made by applying an objective test as to what would be in the person’s best interests. *All the relevant circumstances, including the factors mentioned in the section must be considered, but none carries any more weight or priority than another. They must all be balanced in order to determine what would be in the best interests of the person concerned. The factors in this section do not provide a definition of best interests and are not exhaustive.*^104^

As the guidance notes indicate, it is no mistake that the statutory drafting is so wide open; though in being this, it of course perpetuates the lack of legal certainty of which Shaw was critical when considering the best interests standard at common law. Best interests’ framing reflects the point that the MCA is aimed at being attuned to the particular without much by way of an account of the general. Whilst this does not collapse into simple deference to what a person might wish for (as indicated in the above quotation), decision making under the Act is focused on a specific decision about a specific person at a specific time. To make good on its precepts, it simply could not operate against a set of prescriptive criteria.

The points here are well drawn out in judicial representations of the law that the MCA provides. Three examples may demonstrate the point. First, in *ITW v Z and others*, Munby J emphasises the lack of a hierarchy of considerations within a best interests assessment, that the weight of different factors will be case-specific, and thus that in a given case the judge will decide by reference to whatever they find to be of ‘magnetic importance’.^105^ Applied to the question of how much weight, if any, to give to P’s wishes and feelings (per section 4(6) of the best interests checklist), he goes on to hold that ‘One cannot, as it were, attribute any particular *a priori* weight or importance to P’s wishes and feelings’.^106^ Strikingly, the term ‘*a priori*’ here could be deleted and the sentence instead ended with the words ‘as a matter of law’. Or secondly, in *Wye Valley v Mr B*, Peter Jackson J observes that ‘there is no theoretical limit to the weight or lack of weight that should be given to the person’s wishes and feelings, beliefs and values’.^107^ By analysis, this lack of a theoretical limit of course applies to any factor that might feature in a best interests decision. And again strikingly, the point could be made by changing the phrase ‘no theoretical limit’ to ‘no legal limit’. Or finally, as Hayden J articulated in *M v N and another*, ‘the obligation imposed by statute’ is ‘fully to consider’ the relevant matters.^108^ However, in leading to this conclusion, he describes how the actual ‘factors that fall to be considered in this intensely complex process are infinitely variable’.^109^ In short, *the law*’s guidance is radically limited, while the potential bases of decision making are seemingly unlimited.

A salient follow-up to the observation that the MCA could not serve its own precepts if it enlisted tightly prescriptive criteria might be that adequate predictability can and should be found in the combination of the general but ‘limited’ legal measure with the specific character and circumstances of the person for whom the decision is being made. Yet this does little to account for the investment that section 4 inevitably permits for D’s own views (however carefully these are intended to be dampened), or those of the ‘reasonable’ D. (And this is a point, to revisit my claim in the introduction, that we might equally relate to Hass’s analysis of the (in)capacity test in sections 2 and 3 MCA.^110^) It is widely perceived to be the case within and beyond CoP jurisprudence that *Aintree v James* demands an emphasis on ‘centring’ the actual person P, rather than some abstracted ‘reasonable P’.^111^ Yet, the further (including unspecified) criteria that may (must) be brought into judges’ analysis and the ‘objective’ nature given to the best interests standard mean that there is inevitably an exclusive and all-reaching shroud of the legally unanchored wisdom of the actual decision maker governing the standard’s operation. This point is emphasised in the quoted dictum from Justice La Forest in *Re Eve*, above,^112^ but is also observable in a more rounded analysis of MCA jurisprudence leading up to the present time in England and Wales.^113^

In short, there may be good reason for the best interests standard, as a standard provided in statute, to be incomplete and wide open. But that does not make it good as a *legal* standard, or lend it naturally to ultimate determination by a judge over anyone else (unless judges are, after all, uniquely and best possessed of wisdom).

### C. Radical Departure from the Basics of Common Law Judging

A final radical distinction to challenge mental capacity law and adjudication as being about *law* is found if we consider legal method within MCA adjudication. Again, I quote from Jackson LJ:

The task of the court is to apply the statutory provisions, paying close heed to the language of the statute. Nevertheless, as judges tread their way through this treacherous terrain, it is helpful to look sideways and see how the courts have applied those statutory provisions to other factual scenarios. This has nothing to do with either the doctrine of precedent or the principles of statutory interpretation. The purpose is simply to see how other judicial decisions have exposed the issues or attempted to reconcile the irreconcilable.^114^

Within this statement are several remarkable components. First, Jackson LJ urges attention to the MCA’s words, but not with reference to (my term) ‘standard’ approaches to statutory interpretation. He disavows—because of the particular nature of best interests decisions—the application of a system of precedent; the court is not bound by what has come before (suggesting it was not law), nor is it creating any sort of binding determination (indicating that the decision is not and will not become law). The result for future practice, including adjudication, as Jackson LJ indicates, is not a normative system of action guidance, but a body of generally irreconcilable decisions that (happen to) have been handed down by courts.

Such a vision is consistent with how, in *ITW*, Munby J reconciles the disjuncture between the jurisdiction on the one hand over property and affairs, and on the other over health and welfare.^115^ There, Munby J expressly pronounces more strongly than Lewison J in *Re P*, holding that cases concerning settlements and wills prior to the coming into force of the MCA are ‘consigned to history’.^116^ Taking his phrase from Lewison J, Munby J disavows the applicability of ‘well-known authorities’ and views the courts as instead engaging in ‘the “structured decision making process” prescribed by the 2005 Act’.^117^ Within such a process, a survey of any amount of CoP jurisprudence allows plenty of gleaning *wisdom* by (I repeat here Jackson LJ’s phrase) ‘looking sideways’ to other points of judicial reasoning. Such practice may be exemplified, for instance, by Arbuthnot J where she has *chosen* to make recourse to Cobb J’s alternative best interests checklist, to which I alluded above.^118^ But still, the role of the court comes to seem indistinguishable from the hypothetical non-court that Raz described. Its decision is binding in the instance at hand and enforceable; but it looks to be neither based on nor issued as law. To repeat Lord Bingham’s words (originally advanced in the context of the care and custody of children), ‘it would be very hard indeed to suggest any guideline to govern the exercise of this discretion which was not either so obvious or so heavily qualified as to be futile’.^119^ But this means that we are not and cannot be relying on the judge’s skills in legal method. Rather, the ‘structured decision making process’ of the CoP is one simply requiring judges ‘to show the wisdom, sensitivity and insight of Solomon’.^120^

## 5. Legal Decisions under the Mental Capacity Act 2005: Judicial Power and an Executive Function

The above analysis challenges the substantive essence of the judicial function under the MCA. In legal scholarship on mental capacity law, we find studies that focus on (as it were) the openly non-legal aspects of decisions (eg on the particular values that are or are not identified, and how they are weighted), efforts to argue for how best the MCA ought to operate in practice (ie arguments about the best interpretation of its provisions) or research to cluster or systematise cases against different characteristics or criteria (eg how decisions have been made about withdrawal of life-sustaining treatment), and studies that critique the Act and its adjudication from broader, socio-ethical perspectives (such as by reference to its (in)compatibility with General Statement 1 on Article 12 of the UN Convention on the Rights of Persons with Disabilities). By contrast, what I have sought to do here is identify and scrutinise whatever inner kernels of *law* and *legal* reasoning may be found within judicial determinations where we take the Act and its case law on their own terms. This has allowed a theoretical, but practically oriented, scrutiny of what and how much law features within, or may be drawn from, judgments about what should or should not happen to persons who are deemed unable to make the call for themselves.^121^

My argument is that with the MCA we have a statute and a superabundance of decided cases, but we have a jurisdiction where the law itself is scant. As a normative system to direct action, the regime provided is not exhaustive, nor does it allow clear and binding guidance or predictability. We have a jurisdiction that does not operate according to governance by rules (legal or otherwise). The language of mental capacity law creates empty spaces, and even where it speaks it is highly open textured. As seen above, the Court of Appeal has affirmed that there is no operation of the doctrine of precedent. Rare instances of appeals may overturn a lower court’s decision, but there is no general overruling in favour of generating new substantive rules of law (albeit, as I revisit shortly, there are legal rulings about procedural requirements to govern this system of non-legal decision making). Similarly, there is no standard of interpretation to be made by reference to ‘rules’ of statutory construction. And even in an overarching sense, and even with the enumerated principles in section 1 MCA, it is not a system of governance that is circumscribed by an exhaustive list of values—whether in the sense of a coherent, comprehensive or ordinally ranked list of principles according to which judgment might be made, or in the sense of some in-principle possibility of a singular, overarching political morality from which a ‘Herculean’ judge might draw to find the ‘right’ answer.^122^ Indeed, judges in the CoP are free to devise, refer to or ignore multiple alternative, non-comprehensive checklists. The Act codifies a space to be filled with plural, mutually contradictory sources of values and value conflicts. It is designed that way.

In adjudicating mental capacity cases, what we therefore find is a broad discretionary power afforded to judges. It does not follow that it is arbitrary. But it has at best limited *legal* constraints, and its framing and function radically differ from general understandings of what would be expected of the role of the judicial branch, even where we set a bar with low theoretical and practical demandingness for what ‘law’ should mean, and allow that judging in general requires the exercise of discretion. I would suggest in the context of mental capacity law that the discretion afforded to judges and their role appears more akin to one held in an executive function than a judicial or even a legislative one. Whether or not it is surprising or welcome that the CoP’s workload has become what it is, its function is an administrative reality created by the MCA itself. Insofar as developments have emerged, they have not been, and cannot be, in the generation of substantive law. As Raz hypothesised against his theoretical non-court of law, on substantive matters there is ‘some regularity in the decisions’,^123^ but there is inevitable inconsistency and contingency, and an unavoidably central place for the wisdom of (or at least values identified by) the judge as the determinative factor, emphatically as contrasted with the application of legal rules.

This reflection is sharpened if we consider that the burden of *legal* commands that have emerged in relation to CoP jurisprudence are procedural in nature.^124^ Yet, that again suggests warnings. In being procedurally directed, they operate on the assumption of the CoP serving a judicial rather than an executive role. This creates its own problems given the distinct institutional arrangements and norms at play when we consider the un-lawlike nature of substantive MCA jurisprudence. These problems span more abstract and maybe obvious points, perhaps most notably critiques of the invocation of adversarial proceedings within a process that really demands inquisitorial and, more often than not, collaborative inquiry. And they span more subtle, and related, specific questions, such as on the soundness of applying expectations of ‘standard’ legal procedural propriety in this outlier context.

In approaching the ‘doing’ of mental capacity law, it has been well shown that there is a skills vacuum that cannot be filled, or even mostly filled, through knowledge of the law and the application of legal method: there is a need to attend to what Kong and colleagues label the ‘human element’.^125^ I have suggested in this article that as well as looking outwards towards the human element of MCA decision making, we do well to look (as it were) inwards and revisit critically its pretences to possessing a ‘legal element’. In so doing, I suggest that we find very little that honours the designation ‘law’, which in turn creates problems for the question of the Act’s *legal* adjudication. We must bear this in mind when taking up calls for critical engagement such as that advanced by Sir Mark Hedley, and when moving, in Bartlett’s terms, towards ‘the next generation of law’.^126^ This does not obviously mean that we shun or even limit the place of the CoP within governance of the contexts covered by the MCA (though it may do). But it does invite questions about the provision of a largely uncheckable power, with limited fetters, to any branch of the state where that power’s nature is more executive than it is judicial.

In a bigger picture sense, my analysis thus invites questions about the very ideas of law and sound government.^127^ Against considerations of the place of law and the role of judges in the context of the administrative state, we often find focus on questions of executive versus judicial excess, of institutional competence, of soundly clarified constitutional roles and of distinctions (formal and substantive) in different forms and applications of state power. Analysis of the CoP allows us to advance such arguments by reference to the courts not simply as law-applying, or even law-making, bodies, but also themselves as executive or administrative actors. Even while it should be clearly understood that the overwhelming majority of decision making under the MCA happens outside of the courtroom, under the Act, we see the courts as an operative force *within* the administrative state, rather than a constraining or balancing force against it. Bluntly, this demands that we ask: if the ultimate determination that the MCA allows a judge to make, against standards based in wisdom rather than legal constraint, were placed in the hands of (say) a government minister or a doctor or an administrator within a local authority, should we be content with that? If not, why should we be happy with it falling to judges? To be clear, this is not a rhetorical question. Rather, when considering ‘Where next?’ with the MCA, it is a question that needs directly to be asked and answered.^128^ That answer will have implications too for broader reviews and critiques of the constitutional and institutional role and reach of the judiciary—and, indeed, for the robustness (or otherwise) of understandings of legal positivism or concepts such as ‘hard law’ that pervade legal scholarship.

## 6. Conclusions

How do you stop hard cases from making bad law? One way is to strip their determination of any distinctly *legal* reasoning, and deny that they make *law* at all. In this article, I have set up a way of asking and answering the question ‘Is mental capacity law law?’. I have argued that mental capacity law is not law, and adjudication under the MCA is not legal adjudication. Part of my argument has rested on simple comparison between Joseph Raz’s hypothetical example of an institution that is not a court of law and a reflective but fairly plain description of the MCA and the jurisdiction that it creates. We might be cautious of an apparent body of law and its adjudication when it seems (inadvertently) to have been modelled on a jurisprudential exemplar of what a court of law is not.

In a more engaged sense, I have sought to explain the paradox of affording special privilege to the expert and institutional (and thereby constitutional) position of judges under the MCA: namely, that on the terms set by the Act, the more law-like a judicial decision might be, and the more given it is to the application of legal method, the worse it is. This is because the MCA and its adjudication are about judging values. And while, as a method of practical, normative reasoning, there are overlaps and shared skills in these things, identifying and judging values is not the same as identifying and judging law. Where values are prescribed loosely (as, for example, in human rights instruments), drawn from general principles of law writ large (as, for example, happened in the formation of the tort of negligence) or drawn to reform a patently arcane and brutal legal fiction (as, for example, occurred in relation to spousal rape), we may say that within the function of legal adjudication, any distinction that might be offered between judging values and judging law is one of degree rather than type. And, of course, in such instances the common law judge does not just find and apply law; the judge’s role entails a clear (even if, in its nature and justifiability, disputed) legislative function. With mental capacity law, by contrast, we have an Act of Parliament and body of decided cases that do not just fail to prescribe a complete decision-making framework, but positively rule out its exhaustive provision even through refinement in case law. In other words, we enter a domain that demands a different exercise in practical reason from that which is understood to fall to the judiciary. It is an outright non-legal one. This is only underscored where we find that whatever decision is issued on the back of mental capacity adjudication does not itself become, or even pretend to become, part of ‘the law’. Rather than applying, or even making, law, the MCA has judges serve in an executive function. Against framings of what this means for judicial practice, it places all of the emphasis on practical wisdom and none (or at most vanishingly little) on legal reasoning.

As highlighted through Sir Mark Hedley’s reflections, because of the determinative and enforceable nature of judicial determinations under the MCA, the decision making resides in largely unfettered discretion. Perhaps it reliably calls on judges’ wisdom, but that still becomes legally unchecked wisdom. It is a discretion that one imagines would not be left legally unregulated if prescribed (say) to a minister or local authority. However, I do not close this article inviting the conclusion that this necessarily means that the drafters of the MCA got it wrong. In Sir Mark’s argument, we find what amounts to a twofold defence of the power given to judges: first, some institutional actor has to have the final say; and second, in that event, judges present the best (or least bad) option. That position may be correct. But as well as the scrutiny needed to evaluate that question, accepting the point—which requires acceptance of a constitutional and institutional fudge whose necessity would rest on the higher importance of sound social ethics—invites more fundamental reflection on the nature and normative authority of law itself. And it thereby challenges basic ideas of what it means to engage in distinctly *legal* reasoning, how we can and should understand the division of constitutional and institutional functions of the state, and the place and meaning of the law within that complex system.

## Footnotes

1 Sir Mark Hedley, *The Modern Judge: Power, Responsibility and Society’s Expectations* (LexisNexis 2016) 68–9.

2 The MCA also applies to children aged 16 and 17, but with unequal effect to its application to adults. While the analysis in this article bears on matters regarding children, its focus does not account for wider bodies of law that also apply (notably the Children Act 1989 and the Family Law Reform Act 1969).

3 MCA, ss 3 and 2.

4 See MCA, s 4 for the central aspect of this: the best interests standard. See also ss 9–23 regarding lasting powers of attorney and the appointment of deputies, and ss 24–6 regarding advance decisions to refuse treatment.

5 The best interests standard limits decision making for lasting powers of attorney and court-appointed deputies: see MCA, ss 9(4)(a) and 20(6) respectively. Advance decisions, on their face, permit for effective refusals of treatment against a person’s best interests. However, the qualifications regarding the validity and applicability of advance decisions, combined with direct challenges to the application of rules regarding (in)capacity determinations themselves, create what I would argue to be a *de facto* circumscription by reference to best interests: see MCA, s 25 and the following footnote.

6 Binesh Hass, ‘Reasonableness in Capacity Law’ (2023) 86 MLR 1447. See also Binesh Hass, ‘The Values and Rules of Capacity Assessments’ (2022) 48 Journal of Medical Ethics 816. See also Cressida Auckland, *Values and Disorder in Mental Capacity Law* (CUP 2024). In making these necessarily summary comments, I note that Sir Mark Hedley, who kindly offered comments on an earlier draft of this article, draws a firm distinction between the ‘gateway’ provided by the (in)capacity test in ss 2 and 3 and the consequent best interests jurisdiction that arises in the event of a finding of incapacity. He is of the view that a finding of (in)capacity is ‘a judicial judgment based on proved facts with no obvious discretionary element’. This leads him to the position that findings of incapacity, like the factual threshold criteria under the Children Act 1989, ‘are clearly legal disciplines requiring a formal judicial approach’. As stated, I am more circumspect about this. However, the view demands consideration and respect. As regards the argument of the current article, readers who share Sir Mark’s opinion about (in)capacity assessments will still be able to recognise the argument’s application to the best interests standard, and the questions concerning the role of judges in making these.

7 Prior to the MCA’s coming into force, matters regarding property and affairs were addressed by the Court of Protection of the Supreme Court, while the High Court addressed matters regarding health and welfare under its inherent jurisdiction (and continues to claim and apply powers in this regard with a de-coupling of mental capacity and decision-making capacity: see Michael Dunn, Isabel Clare and Anthony Holland, ‘To Empower or Protect? Constructing the “Vulnerable Adult” in English Law and Public Policy’ (2008) 2 LS 234).

8 Even if the MCA fares better against challenges from a perspective afforded by the European Convention on Human Rights, its consistency with the UN Convention on the Rights of Persons with Disabilities is (to put it mildly) questionable. See generally Peter Bartlett, ‘Re-thinking the Mental Capacity Act 2005: Towards the Next Generation of Law’ (2023) 86 MLR 659.

9 ibid. See also Law Commission, *Mental Capacity and Deprivation of Liberty* (Law Com No 372, 2017); Rosie Harding, ‘Safeguarding Freedom? Liberty Protection Safeguards, Social Justice and the Rule of Law’ (2021) 74 CLP 329; Lucy Series, *Deprivation of Liberty in the Shadows of the Institution* (Bristol UP 2022). It might be noted that the Mental Capacity (Amendment) Act 2019, enacted consequent to the Law Commission report cited here, is still not in force at the time of writing.

10 Sir Mark Hedley’s book *The Modern Judge* (n 1), from which the quotation at the head of this article draws, looks as well at criminal sentencing, child welfare and disputes about medical treatment. Perhaps given the framing that I offer, we might relate these ideas to literatures on public law and the application of human rights law, and the proper role of judges. This must not be done with disregard for the significant qualitative distinctions between private individuals being the subject of legally coerced decisions made on their behalf and public bodies being constrained by law. There should not be disregard either for the reach of such discussions into areas of private law: see eg Paul Craig, ‘Limits of Law: Reflections from Private Law and Public Law’ in NW Barber, Richard Ekins and Paul Yowell (eds), *Lord Sumption and the Limits of the Law* (Bloomsbury 2016). See also Binesh Hass’s comments as regards the problems of applying public law’s *Wednesbury* standard within the context of personal decision-making: Hass, ‘Reasonableness in Capacity Law’ (n 6).

11 The weight and nature of powers under the MCA are sometimes understated or distinguished from the more ostensible application of coercive force under the Mental Health Act 1983. For a compelling, critical analysis, see Lucy Series, ‘Of Powers and Safeguards’ in Atina Krajewska and Jean McHale (eds), *Re-Imagining Health and Care Law* (Edward Elgar Publishing, forthcoming) (for pre-print <https://research-information.bris.ac.uk/ws/portalfiles/portal/357733004/Series_2023_Of_power_and_safeguards_pre_print.pdf>).

12 Sir Denzil Lush notes the Court of Protection’s ‘origins go back to at least the second half of the thirteenth century, when the crown assumed responsibility for the estates of lunatics and idiots, and this jurisdiction was certainly in place by 1324, when the Statute *de Praerogativa Regis* confirmed its continuation’. Denzil Lush, ‘The Mental Capacity Act and the New Court of Protection’ [2005] Journal of Mental Health Law 31, 32.

13 Bartlett (n 8).

14 Law Commission, *Mental Incapacity* (Law Com No 231, 1995).

15
*Re F (Mental Patient: Sterilisation)* [1990] 2 AC 1.

16 ibid 52.

17 ibid.

18 We also find the development of what might be labelled ancillary common law rules, such as a clarification that under no circumstance could legal power arise to allow consent on behalf of the person (see *Airedale NHS Trust v Bland* [1993] AC 789) or surrounding the potential for a person to engage in anticipatory decision making through provision of an advance decision (see *HE v A Hospital NHS Trust* [2003] 2 FLR 408).

19
*Re C (Refusal of Medical Treatment)* [1994] 1 FLR 31.

20 See *Bland* (n 18).

21 Contrast eg the reasoning across the judgments in ibid. See also John Coggon, ‘Legal Expertise and Its Subject Matter within Common Law Adjudication’ in Mirko Farina and Andrea Lavazza (eds), *Philosophy, Expertise, and the Myth of Neutrality* (Routledge 2024).

22 cf eg *Re A (Mental Patient: Sterilisation)* [2000] 1 FLR 549; *Re S (Adult Patient: Sterilisation: Patient’s Best Interests)* [2001] Fam 15.

23 MCA, s 1(2).

24 MCA, s 3 provides a functional test for incapacity, to be followed, if incapacity is indicated, by a diagnostic test in s 2. On the operation and application of the test, see *PC v City of York Council* [2013] EWCA Civ 478; [2014] 2 WLR 1, [58]–[59] (Macfarlane LJ).

25 In contrast with the previous position at common law, the MCA allows decision-making power to be conferred to third parties for health and welfare decisions as well as for property and affairs, and formalises provisions regarding advance decision making: see n 4 above. As noted in the introduction to this article, with the (arguable) exception of advance decisions, the governance of these matters is circumscribed by the best interests standard.

26 MCA, s 4(6).

27 MCA, s 4(2).

28 MCA, s 4(11).

29 The article’s analytical jurisprudential critique adds to a voluminous literature that exhibits various methodological approaches and critical perspectives, which focus in particular on the predominance of institutional power and political liberal norms, and their shaping of the (apparent) law that governs personal decision making. For just a small representation, see Mary Donnelly, *Healthcare Decision-Making and the Law: Autonomy, Capacity and the Limits of Liberalism* (CUP 2010); Camillia Kong, *Mental Capacity in Relationship: Decision-Making, Dialogue, and Autonomy* (CUP 2017); Jonathan Herring, *Law and the Relational Self* (CUP 2019); Beverley Clough, *The Spaces of Mental Capacity Law: Moving Beyond Binaries* (Routledge 2022); Mary Donnelly, Rosie Harding and Ezgi Tascioglu (eds), *Supporting Legal Capacity in Socio-Legal Context* (Hart 2022); Jaime Lindsey, *Reimagining the Court of Protection: Access to Justice in Mental Capacity Law* (CUP 2022); Auckland (n 6).

30 Perhaps the single most useful resource is 39 Essex Chambers’ Mental Capacity Resource Centre <www.39essex.com/information-hub/mental-capacity-resource-centre>.

31 Department for Constitutional Affairs, *The Mental Capacity Bill: Full Regulatory Impact Assessment* (2005) para 43.

32 Lucy Series and others, ‘Welfare Cases in the Court of Protection: A Statistical Overview’ (Cardiff University 2017).

33 Bartlett (n 8).

34
*Aintree University Hospital NHS Foundation Trust v James* [2013] UKSC 67, [2014] AC 591.

35 Contrast the approach taken by Lord Justice Peter Jackson, ‘Is Family Law Law?’ (The Nicholas Wall Memorial Lecture, 11 May 2023) <www.judiciary.uk/the-nicholas-wall-memorial-lecture-given-by-lord-justice-peter-jackson-is-family-law-law/>.

36 cf Paul Craig, ‘Formal and Substantive Conceptions of the Rule of Law: An Analytical Framework’ [1997] PL 467; John Tasioulas, ‘The Rule of Law’ in John Tasioulas (ed), *The Cambridge Companion to the Philosophy of Law* (CUP 2020) ch 6.

37 Joseph Raz, *The Authority of Law: Essays on Law and Morality* (Clarendon Press 1979) ch 11.

38 ibid 218 (emphasis added).

39 ibid 214 (emphasis in original).

40 ibid 214.

41 ibid 218.

42 ibid 214–19.

43 ibid (reproducing verbatim each of the headings for the principles that Raz gives).

44 ibid 219 (emphasis added).

45 ibid fn 8.

46 cf Tom Bingham, *The Business of Judging* (OUP 2000) ch 3.

47 Canonically, see HLA Hart, *The Concept of Law* (2nd edn, OUP 1994).

48 eg *R (Quintavalle) v Secretary of State for Health* [2003] UKHL 13, [2003] 2 WLR 692.

49 eg *R v R* [1991] UKHL 12, [1992] 1 AC 599.

50 eg *Donoghue v Stevenson* [1932] AC 562.

51 See Neil Duxbury, *The Nature and Authority of Precedent* (CUP 2008).

52 eg Hart (n 47).

53 eg Rachel Cahill-O’Callaghan, *Values in the Supreme Court: Decisions, Division and Diversity* (Hart Publishing 2020).

54 eg Barber, Ekins and Yowell (n 10).

55 When presenting an earlier version of this article at the University of Oxford on 23 November 2022, I was asked whether I was essentially just arguing for a legal realist position. That is not, I suggest, a sound reading of my position. A legal realist would possibly share (something like) my view of mental capacity law adjudication. But crucially, the argument I advance in this article is not a legal realist’s one. Rather, it is that (at best) only the thinnest pretences to applying law are available to judges in MCA cases. Furthermore, by its nature, the analysis in this article is about a *specific* jurisdiction that differs from the general, rather than a *general* representation of the facts, processes or motivations that are at play in adjudication writ large. In short, theorists who reject legal realism could coherently do so whilst still accepting the arguments in this article; and even analysts who buy wholesale into legal realism may see a different practical exercise in judges’ provision of reasons in mental capacity law cases to those in other cases that they study.

56 Raz (n 37) 48.

57 Lord Judge, ‘Reflections of a Retired Lord Chief Justice’ in Jeremy Cooper (ed), *Being a Judge in the Modern World* (OUP 2017) 20.

58 <www.judiciary.uk/about-the-judiciary/our-justice-system/three-is/>.

59 Lord Reid, ‘The Judge as Law Maker’ (1972–73) 12 JSPTL 22.

60 cf eg John Finnis, ‘The Fairy Tale’s Moral’ (1999) 115 LQR 170; Allan Beever, ‘The Declaratory Theory of Law’ (2013) 33 OJLS 421; Lord Mance, ‘Should the Law Be Certain?’ (The Oxford Shrieval Lecture, 11 October 2011) <https://supremecourt.uk/speeches/lord-mance-at-the-oxford-shrieval-lecture>.

61 Raz (n 37) 111–12.

62 ibid 112.

63 Josephine Shaw, ‘Sterilisation of Mentally Handicapped People: Judges Rule OK?’ (1990) 53 MLR 91.

64 Margaret Brazier and José Miola, ‘Bye-Bye Bolam: A Medical Litigation Revolution?’ (2000) 8(1) Med L Rev 85.

65 A great part of Shaw’s critique leads to an invitation for parliamentary intervention, which did, of course, eventually happen in the form of the MCA: but one wonders, given what I argue below, whether the benefits that Shaw perceived in the creation of primary legislation really materialised with the statute.

66
*Re Eve* (1987) 31 DLR (4d) 1, 32.

67
*Bland* (n 18) 887.

68 Hedley (n 1) 49.

69 MCA, s4(2).

70 Hedley (n 1) 49.

71 ibid 47.

72 Bingham (n 46) 49.

73 ibid.

74 On which see the judicial reflections in eg *Re P* [2009] EWHC 163 (Ch), [2009] 2 All ER 1198; *ITW v Z and others* [2009] EWCOP 2525. Both cases are discussed further below.

75
*Cambridge University Hospitals NHS Foundation Trust v BF* [2016] EWCOP 26, [29].

76 On the idea of P-centricity, see Camillia Kong, ‘The Phenomenology and Ethics of P-Centricity in Mental Capacity Law’, (2023) 42 Law and Philosophy 145.

77
*Aintree v James* (n 34) [36].

78 See further John Dewar, ‘The Normal Chaos of Family Law’ (1998) 61 MLR 467 and the debates that paper has provoked. See also Jackson (n 35).

79 cf Lush (n 12).

80 Kong, ‘The Phenomenology and Ethics’ (n 76). See also Camillia Kong, *Mental Capacity in Relationship* (n 29).

81
*NHS North Central London Integrated Care Board v Royal Hospital for Neuro-Disability and another* [2024] EWCOP 66 (T3), [59].

82
*PL v Sutton Commissioning Group and another* [2017] EWCOP 22.

83 ibid [9].(iii).

84 ibid [9].(iii)(g).

85 ibid [9].(iii)(f).

86 See John Coggon and Camillia Kong, ‘From Best Interests to Better Interests? Values, Unwisdom and Objectivity in Mental Capacity Law’ (2021) 80 CLJ 245, 250–4.

87
*Re P* (n 74) [36] (emphasis added).

88
*W v M & Ors* [2011] EWHC 2443 (Fam), [2011] EWHC 2443 (Fam), [85] (emphases added).

89
*Aintree v James* (n 34); *Briggs v Briggs and others* [2016] EWCOP 53.

90
*Re S* (n 22).

91
*Aintree v James* (n 34) [24].

92 cf Søren Holm and Andrew Edgar, ‘Best Interest: A Philosophical Critique’ (2008) 16 Health Care Analysis 197.

93
*RB v Brighton and Hove City Council* [2014] EWCA Civ 561, [2014] CN 854, [40].

94
*Re S* (n 22) 27–8.

95
*Re S (Hospital Patient: Court’s Jurisdiction)* [1995] Fam 26, 32.

96
*Aintree v James* (n 34) [40]–[42].

97 Camillia Kong and others, ‘An Aide Memoire for a Balancing Act? Critiquing the “Balance Sheet” Approach to Best Interests Decision-Making’ (2020) 28 Med L Rev 753.

98
*Re N* [2015] EWCOP 76, [70].

99 ibid.

100
*Briggs v Briggs* (n 89) [62].

101
*Aintree v James* (n 34) [23].

102 cf eg *Wye Valley NHS Trust v Mr B* [2015] EWCOP 60, [8] (Jackson J); *Re AB* [2019] EWCA Civ 1215, [28] (King LJ, with whom McCombe and Peter Jackson LLJ agreed).

103 Raz (n 37) 111–12.

104 ‘Explanatory Notes: Mental Capacity Act 2005’ para 28 <www.legislation.gov.uk/ukpga/2005/9/notes> (emphases added).

105
*ITW v Z and others* (n 74) [32].

106 ibid [35].

107
*Wye Valley v Mr B* (n 102) [8].

108
*M v N and another* [2015] EWCOP 9, [28].

109 ibid.

110 See n 6 above.

111 See eg *Avon and Wiltshire Mental Health Partnership and another v WA and others* [2020] EWCOP 37, [48] (Hayden J).

112 See text to n 66 above.

113 See Coggon and Kong (n 86).

114
*RB v Brighton and Hove City Council* (n 93) [40].

115 See text to n 87 above.

116
*ITW v Z and others* (n 74) [29].

117 ibid.

118 See eg *Mid-Yorkshire Hospitals NHS Trust v NB and another* [2022] EWHC 3682 (Fam), [45]; *King’s College Hospital NHS Foundation Trust v XY and another* [2024] EWCOP 68, [100].

119 Bingham (n 46) ch 3.

120 ibid.

121 My focus has been on best interests decisions, but, as suggested in the introduction, I would argue that the points apply with equal force to determinations of incapacity and—because of how they are presented in the MCA—circumscribes decision making by legally empowered third parties or through advance decisions.

122 cf Ronald Dworkin, *Law’s Empire* (Harvard UP 1988).

123 Raz (n 37) 111–12.

124 Consider the reasoning and effect, for instance, in the Court of Appeal decision in *Re AH* [2021] EWCA Civ 1768.

125 Camillia Kong and others, ‘The “Human Element” in the Social Space of the Courtroom: Framing and Shaping the Deliberative Process in Mental Capacity Law’ (2022) 42 LS 715.

126 Bartlett (n 8).

127 Such questions are well explored alongside distinct but complementary critiques of ‘reimagining’ law and government, and the roles and reach of organs of the state, through the lens of other complex, applied areas: eg Elizabeth Fisher, ‘Executive Environmental Law’ (2020) 83 MLR 163.

128 I do not attempt in this article to advance a view on what, if anything, would be a better model. For a discussion and proposal in this regard, applied to determinations of (in)capacity, see Carina Schacherl and Alex Ruck Keene, ‘Determining Capacity—Are Three Heads Better Than One?’ (2010) 1 Journal of Elder Law and Capacity 11.

